# P-2116. Analysis of No-Growth Wells in *Escherichia coli* (EC) During Fosfomycin Broth Microdilution (BMD) and Agar Dilution (AD) Testing

**DOI:** 10.1093/ofid/ofae631.2272

**Published:** 2025-01-29

**Authors:** Jenna Salay, Khadijah Malik, Lindsey Collins, Tiffany Chang, Morgan L Bixby, Morgan L Bixby, Elizabeth B Hirsch

**Affiliations:** University of Minnesota College of Pharmacy, Minneapolis, Minnesota; University of Minnesota College of Pharmacy, Minneapolis, Minnesota; University of Minnesota College of Pharmacy, Minneapolis, Minnesota; University of Minnesota, Twin Cities, Minneapolis, Minnesota; Univeristy of Minnesota, Saint Paul, Minnesota; Univeristy of Minnesota, Saint Paul, Minnesota; University of Minnesota College of Pharmacy, Minneapolis, Minnesota

## Abstract

**Background:**

Although AD is the reference method for CLSI and EUCAST, it is not clinically efficient. Neither CLSI nor EUCAST approve the use of BMD due to less precision, trailing endpoints, and presence of no-growth wells before the minimal inhibitory concentration (MIC). However, available data describing these phenomena are very limited. We sought to evaluate rates of scientific error—no-growth wells and growth at concentrations above the MIC in BMD and AD—among a collection of EC isolates, to determine if BMD is truly less precise than AD.

**Figure:**
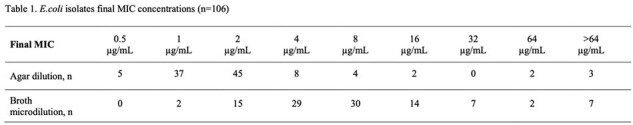

**Methods:**

A convenience collection of EC clinical urine isolates (n = 106) collected in 2022 was included. BMD and AD were performed in technical triplicate and in biological duplicate on separate days to determine a final MIC. A measured MIC was defined as the MIC obtained from a single biological replicate whereas the final MIC was the shared MIC (± 1 dilution) between two biological replicates of the same isolate. For AD, MIC were determined from the first concentration with no growth or only a single colony formed; for BMD: lowest concentration with no visible growth in all three technical replicates. For BMD, a no-growth well was defined as lack of growth at any concentration below the MIC. Growth above the measured MIC was defined as a single well with any visible growth at a concentration greater than the MIC. For AD, error was recorded as any single colonies at the MIC or any countable colony growth at concentrations above the MIC.

**Figure:**
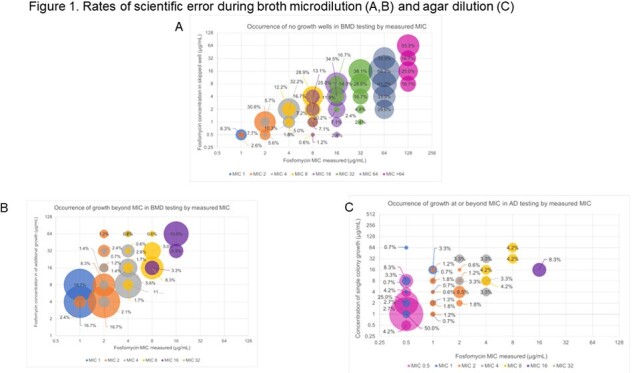

**Results:**

The collection had a range of final MIC from 0.5 to > 64 µg/mL for AD and BMD (Table 1). For BMD final MIC including data for > 2 isolates, no-growth wells occurred up to 38.1% of the time (Figure 1A). Additional growth beyond the MIC occurred up to 16.7% of the time (Figure 1B). Single colonies at or beyond the MIC occurred up to 50% of the time (Figure 1C).

**Conclusion:**

Rates of no-growth wells and growth at concentrations higher than the measured MIC for fosfomycin BMD and AD testing was high among a collection of EC isolates, which resulted in frequent scientific error. BMD had increased rates of scientific error while reading results. Additional analysis using a larger isolate set is needed to further validate these results; however, the frequency of error demonstrated for both testing methods should prompt re-evaluation of fosfomycin BMD and AD testing.

**Disclosures:**

Elizabeth B. Hirsch, PharmD, FCCP, FIDSA, GSK: Advisor/Consultant|GSK: Honoraria

